# Clumped isotopes reveal relationship between mussel growth and river discharge

**DOI:** 10.1038/s41598-024-58246-w

**Published:** 2024-04-01

**Authors:** Melanie A. Brewer, Ethan L. Grossman, Charles R. Randklev

**Affiliations:** 1 Department of Geology and Geophysics, Texas A&M University, College Station, TX 77843 USA; 2 Texas A&M Natural Resources Institute, Dallas, TX 75252 USA

**Keywords:** Ecology, Environmental sciences, Hydrology

## Abstract

Freshwater mussels preserve valuable information about hydrology, climate, and population dynamics, but developing seasonal chronologies can be problematic. Using clumped isotope thermometry, we produced high-resolution reconstructions of modern and historic (~ 1900) temperatures and δ^18^O_water_ from mussel shells collected from an impounded river, the Brazos in Texas, before and after damming. We also performed high-resolution growth band analyses to investigate relationships between mussel growth rate, rainfall, and seasonal temperature. Reconstructed δ^18^O_water_ and temperature vary little between the modern (3R5) and historic shell (H3R). However, a positive relationship between reconstructed δ^18^O_water_ and growth rate in H3R indicates that aside from diminished growth in winter, precipitation and flow rate are the strongest controls on mussel growth in both modern and pre-dam times. Overall, our results demonstrate (1) the impact, both positive and negative, of environmental factors such as flow alteration and temperature on mussel growth and (2) the potential for clumped isotopes in freshwater mussels as a paleohydrology and paleoclimate proxies in terrestrial environments.

## Introduction

Freshwater mussels (Order: *Unionida*) are arguably the most threatened group of animals in the world^1,2^, with nearly 70% of mussels in North America alone considered extinct or imperiled, and the remaining mussels living within fragile environments in need of urgent protection and proactive conservation^3^. Threats from habitat destruction, over-exploitation, water quality degradation, invasive species, and climate change are adding immense pressure to mussel survival, reproduction, and dispersal. Habitat destruction can be caused by alteration of natural flow regimes resulting from river impoundment, stream channelization, increases in impervious surfaces, ground water withdrawals, and changes in land use practices^4–6^. These environmental and anthropogenic stressors are associated with the mussel declines seen in the past and present, with alteration to natural flow regime being one of the most important because it shapes channel morphology, regulates water temperature, and influences water quality and nutrient cycling, which taken together determine mussel habitat in rivers and streams^7–10^. Thus, changes in flow that affect any one of these factors can result in shifts or elimination of mussel habitat, which over time can drive changes in species occurrence and community composition^4^.

Unfortunately, the causal mechanisms underpinning these community level changes remain largely unknown. This is due in large part to the lack of information on how population performance (i.e., growth, survivorship, and reproduction) is affected by changes to flow and temperature. A notable exception is the study by Rypel et al.^11^, who looked at the effect of river impoundment on shell growth in select streams in the southeastern United States. The authors found that in regulated streams, mussel growth had become decoupled from hydrologic variation, but the long-term implication of their findings is unclear. Bolotov et al.^12^ evaluated the effects of climate change on margaritiferid mussels, a sister family to unionids, using shell morphology and growth rates, finding that a climate warming of ~ 6 °C in mean summer temperature over the past 100 years likely contributed to their decline. These findings demonstrate the utility of using shell growth as means to evaluate the relationship between mussel growth, flow rates, and climate change.

Using shells to estimate age, growth, and mussel-environment relationships is rooted in the idea that shell growth is slower during the winter and faster in spring and summer but can be dampened or accelerated by environmental stress and certain anthropogenic practices^13,14^. However, these growth patterns can vary from year to year, and generally show an inverse relationship with age where shell growth slows as the organism ages^15,16^. Within the shell, slower growth during the winter season has typically been recognized as dark “annual” bands that are prominent on both the shell exterior and within cross-section^17,18^. Yet, similar banding has also been shown to occur in other seasons as a response to disturbance (i.e., sudden change in temperature, entrainment during floods, handling by surveyors, etc.) and can lead to misinterpretation of seasonality, growth rate, and age in individuals^4^. Therefore, accurately determining these bands is critical for reconstructing and interpreting the ecological and environmental records within freshwater mussels that can live multiple decades to more than a century^16,19,20^. Annual bands in sclerochronology studies have been confirmed with the help of δ^18^O (^18^O/^16^O) thermometry of shells^15,21–25^. However, in many freshwater systems, especially in the impounded river systems commonly found throughout the U.S., decoupling of the temperature and water δ^18^O (δ^18^O_water_) signals makes relating annual bands to cooler δ^18^O temperatures problematic^26–28^, rendering a second unambiguous method for determining sclerochronologies critical for reconstructing and interpreting the ecological and environmental records in the shells. One such method is clumped isotopes.

Clumped isotopes, the incorporation of two rare isotopes in a molecule, has become one of the most important developments in low-temperature geochemistry in the last two decades^29^. Clumped isotope analyses (Δ_47_) of carbonates examines the bonding, or “clumping”, between ^13^C and ^18^O isotopes in carbonate molecules and their deviation from the stochastic distribution. Clumping is enhanced by thermodynamic stability of the ^13^C–^18^O bond and decreases as temperature increases^30^. Unlike conventional oxygen isotope thermometry, where isotopic compositions are dependent on both temperature and δ^18^O_water_^31^, clumped isotope thermometry is strictly temperature dependent, making it an ideal tool for estimating growth temperatures and establishing chronologies^27,32^. In addition, knowing the δ^18^O of the carbonate and Δ_47_ temperature, δ^18^O_water_ can be calculated from the rearrangement of the conventional oxygen isotope thermometry equation (e.g.,^33,34^).

The application of clumped isotopes has been utilized in numerous marine studies for reconstructing paleoclimate and δ^18^O_water_ in ancient oceans (e.g.,^35–39^, but only recently applied to fluvial environments and freshwater mussel shells, confirming their utility as paleohydrologic proxies^27^. In river systems, the reconstructed δ^18^O_water_ and Δ_47_ temperature provide critical information for understanding the cycling of water, precipitation, and evaporation. Compared with marine environments, rivers are generally more variable due to interruptions in hydrologic connectivity, variability of the input source, and greater seasonal temperature variation. Van Plantinga and Grossman^27^ found that Δ_47_ values produced reliable temperatures and reconstructed δ^18^O_water_ values that were within measured river values. However, the resolution was low because of the small number of analyses and large sample size requirements at the time.

In our study, we combine high-resolution clumped isotope analyses with near daily-resolution growth band analyses to produce temperature, δ^18^O_water_, and growth rate records of monthly to bi-monthly resolution in *Amblema plicata,* common name Threeridge (hence “3R” in specimen ID). The threeridge mussel is a common mussel distributed in a broad range of rivers, streams, and lakes within central and eastern North America^3^. We compare our reconstructed δ^18^O_water_ values to measured river data from Van Plantinga and Grossman^40^ and local precipitation data (1) to demonstrate that clumped isotopes can serve as a high-resolution proxy for reconstructing local rainfall and drought events in terrestrial environments, and (2) to evaluate the role of seasonality and rainfall in controlling mollusk growth rates. This information is critical to better understanding river hydrology, climate, and mussel ecology in prehistoric, historic, and anthropogenic times.

### Study area and climate setting

**Figure 1:**
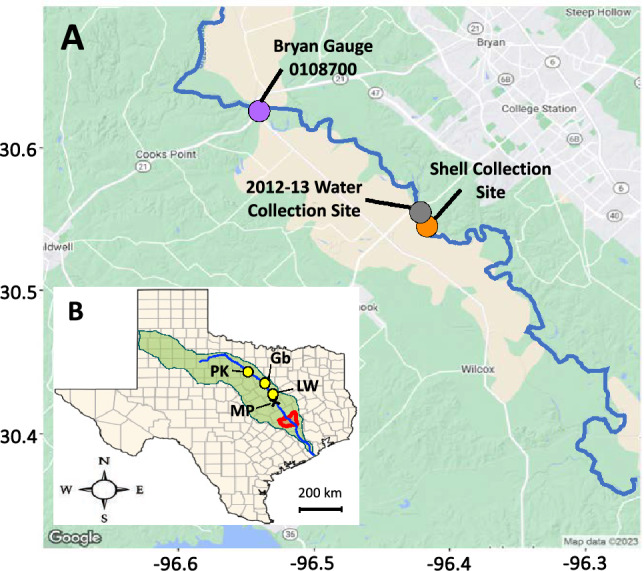
(**A**) Map of the study area showing the Bryan discharge and elevation monitoring gauge (purple), water sampling site (gray), and shell sampling site (orange). (**B**) Inset map showing the study area in the Brazos River, TX drainage basin in green shading, river path in solid blue line, and study area in red shading. Yellow points represent the 3 major dams along the Brazos: Possum Kingdom (PK); Lake Granbury (Gb); and Lake Whitney (LW) dams. Black “x” represents the sampling site for δ^18^O in rainwater during 1962–65 and 1972–76. Note: Maps were created in R v4.3.1 and Rstudio v2023.− 9.1 + 494 using the ggmap and tidyverse packages with the roadmap satellite image sourced from Google Maps https://www.google.com/maps/@30.5656804,-96.4597714,11.57z?entry=ttu and the map_data [in ggplot2] function with in-built datasets. Points and shading on maps were added using ggplot2 and Microsoft PowerPoint v16.81, respectively**.**

The study area is the main stem of the middle Brazos River in Texas (Fig. 1), where mussel shells were collected outside of College Station in ~ 1900 and 2013. The modern shell was collected live from the shoal waters of the Brazos River. The historic shell lacks detailed information about the collection site. However, both shells were collected at almost the same location, which is represented by the marker labelled “Shell Collection Site” in Fig. 1A.

The Brazos River basin is the second largest watershed in Texas and spans the longest distance for a river located entirely in Texas^41^. The river itself is often referred to as the boundary between arid west Texas, where evaporation often exceeds precipitation, and the more humid east Texas that receives more rain. The region’s water is supplied by precipitation, groundwater, numerous smaller tributaries and creeks, and impoundments of upstream dams.

Climate within the study area is temperate with average monthly air temperatures of 13 °C in winter to 29 °C in summer, and an annual rainfall of 89–102 cm with peak precipitation in May and September^42,43^. Air temperature and water temperature within the middle Brazos generally follow each other closely with 1–2 °C variation and a delay on the scale of hours to days depending on the water depth (Fig. S7;^44,45^). Groundwater recharge in region tends to be confined to localized portions of the river and is primarily sourced from recent event waters (i.e., flooding and precipitation) stored in the stream banks. Previous works have found that the river gains groundwater discharge from the unconfined Brazos River Alluvium during high river discharge and can receive deeper groundwater discharge from the Yegua-Jackson aquifer during low river discharge as river flow increases^46–48^. Both ground water discharge and release from impounded reservoirs, primarily from the upstream Lake Whitney^40,47^, can buffer the river temperature by providing sources of cool water in the summer and warm water during the winter^49^. However, shells were collected away from potential influences (i.e., impoundments and water treatment release) that may contribute to short term instability in the air and water temperature relationship, so for the purpose of this study, we can assume that monthly air temperature serves as a reasonable representation of mean monthly water temperatures.

## Results

**Figure 2:**
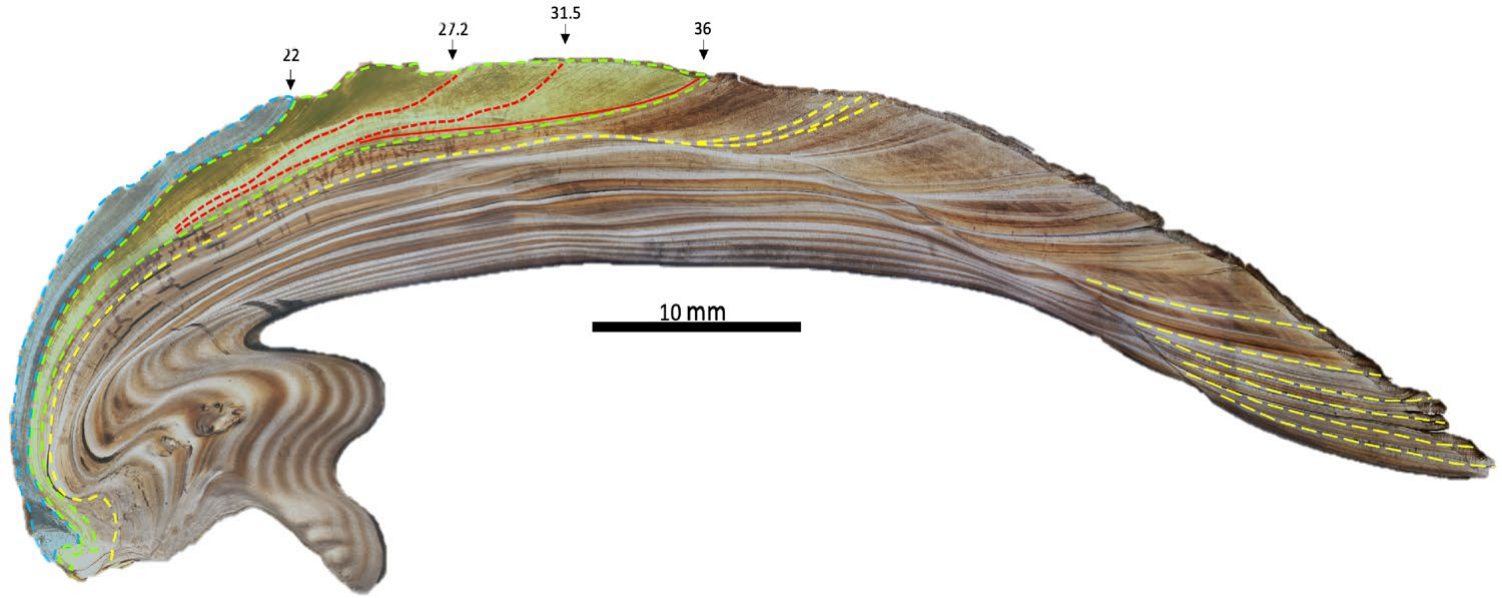
Transmitted light image of specimen H3R (*Amblema plicata*) with sampling area in shaded in blue and green to represent growth for year 1 and 2, respectively. Red lines show bifurcation in opaque “annual band” towards the ventral margin. Yellow dashed lines show additional annual bands not sampled.

Microscopic examination of thin sections allows the discrimination of dark and light bands in the shell cross-sections, the first step in developing sclerochronologies. Dark bands are visible under transmitted light as grey bands relative to other growth periods that appear light brown to tan. Generally, dark bands continue throughout the shell as a single band. However, in the juvenile portion of our specimens, some dark bands start as a single band and bifurcate into multiple dark bands near the ventral margin of the shell (Fig. 2).

**Figure 3:**
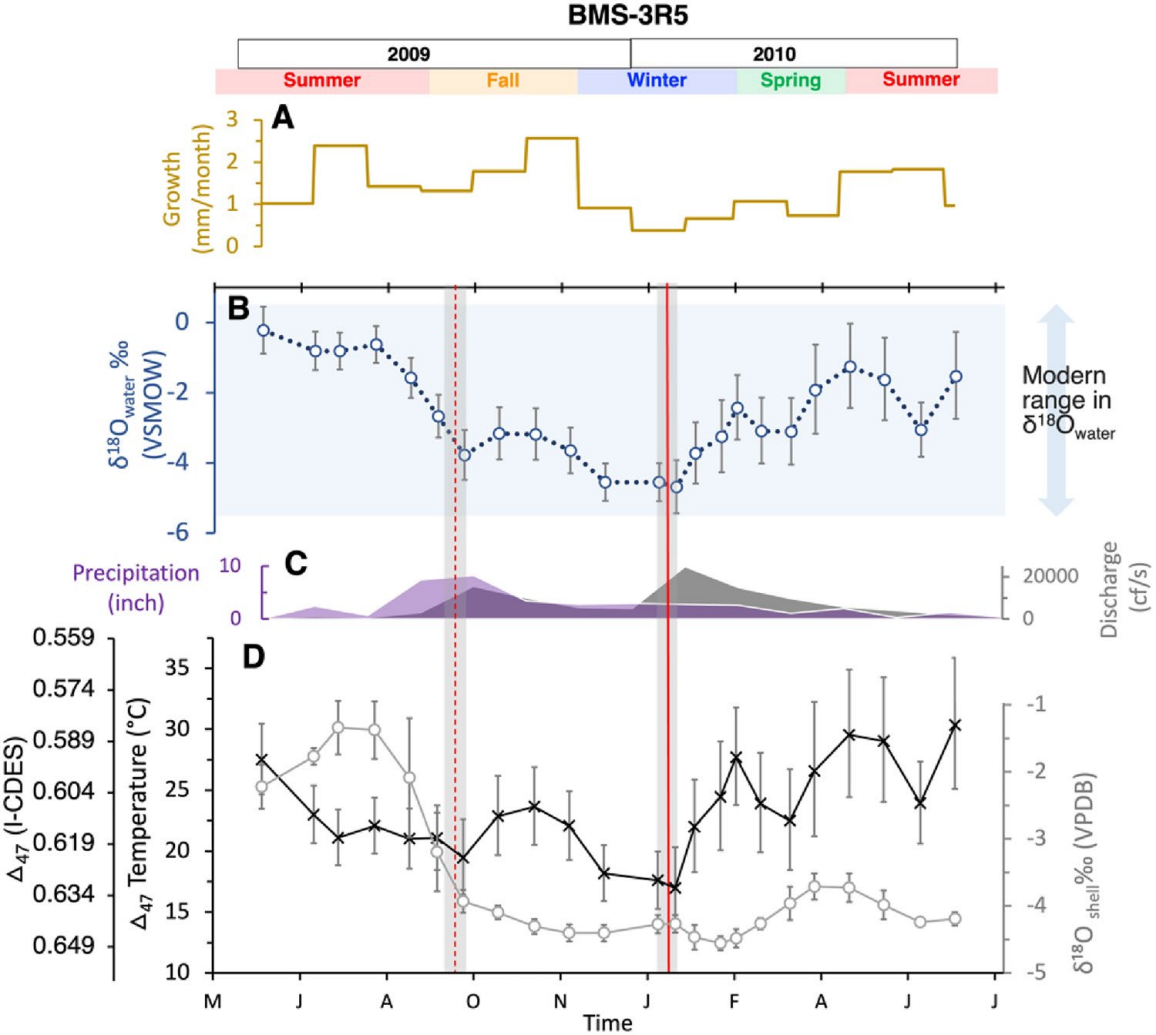
Data for modern shell 3R5. From top to bottom: (**A**) monthly growth rate (yellow); (**B**) reconstructed δ^18^O_water_ (blue); (**C**) measured precipitation (purple) and discharge (dark grey); and (**D**) shell δ^18^O (light grey) and △_47_ temperature (black). The vertical shaded bands represent dark bands in the shell, with the dashed red line representing a disturbance band and the solid red line representing an annual band in the 3B-D. Modern measured values of δ^18^O_water_ are represented by the light blue horizontal rectangle in 3B. Seasons and years are shown at the top.

**Figure 4:**
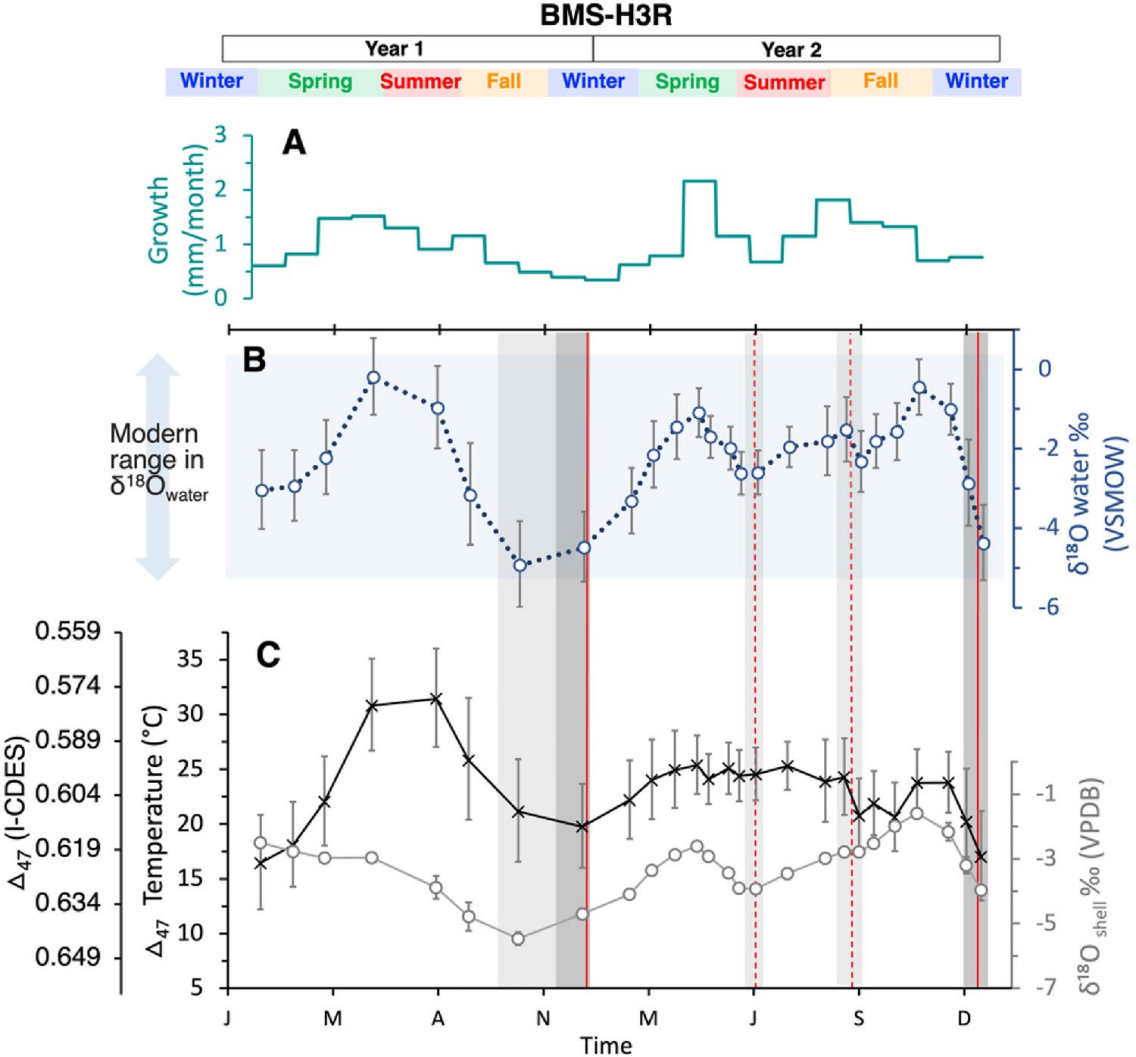
Data for historic shell H3R. From top to bottom: (**A**) monthly growth rate (teal); (**B**) reconstructed δ^18^O_water_ (blue); (**C**) shell δ^18^O (light grey) and △_47_ temperature (black). The vertical shaded bands represent dark bands in the shell, with the dashed red line representing a disturbance band and the solid red line representing an annual band in the 4B-C. Modern measured values of δ^18^O_water_ are represented by the light blue horizontal rectangle in 4B. Seasons and years are shown at the top.

Our reconstructed sclerochronologies have nearly monthly resolution in the first year’s growth and are extended to bi-monthly (two samples per month) in the second year as the shell became longer and sampling area extended. Viewed from bottom to top to best show the sequence of analyses, Figs. 3 and 4 show the progression of measured δ^18^O_shell_ and calculated Δ_47_ temperatures (T(Δ_47_); Figs. 3D and 4C), comparison with available discharge records (Fig. 3C), and reconstructed δ^18^O_water_ (Figs. 3B and 4B) and monthly growth rate (Figs. 3A and 4A). Measured δ^13^C and δ^18^O in modern shell 3R5 show no significant correlation (Fig. S3B) and range from − 10.9 ± 0.6‰–− 5.2 ± 0.2‰ to − 4.6 ± 0.1‰– − 1.3 ± 0.4‰, respectively (Table S1). Measured δ^13^C and δ^18^O in historic shell H3R show a significantly negative trend (Fig. S3B) and range from − 8.9 ± 0.1‰– − 6.7 ± 0.2‰ to − 5.5 ± 0.2‰– − 1.6 ± 0.1‰, respectively (Table S1).

The Δ_47_ values for 3R5 and H3R range from 0.579 ± 0.015 to 0.619 ± 0.012 and 0.575 ± 0.0012 to 0.620 ± 0.013, respectively. T(Δ_47_) values are within monthly mean temperatures of 10.8 °C in January to 29.8 °C August (Fig. S4;^35^) and previous mussel tolerances of ~ 12 to 35 °C^2,36–38^. T (Δ_47_) ranges from 17 ± 3 to 30 ± 5 °C in 3R5 (Fig. 3D) and 16 ± 4 to 31 ± 5 °C in H3R (Fig. 4C). Preliminary chronologies were established following the trend for monthly mean temperature data for College Station (Fig. S6), where lowest and highest clumped isotope temperatures are assigned to the months with minimum and maximum recorded temperature, January and July, respectively. The lowest temperatures were measured in the dark bands that cut through both the interior and exterior surface of the shell and confirm previous studies of “annual banding” in freshwater mussels^11,21,50^*.* Dark bands that failed to cut throughout the outermost layer in the shell were determined to be disturbance bands and occurred at both cool and warm temperatures.

**Figure 5:**
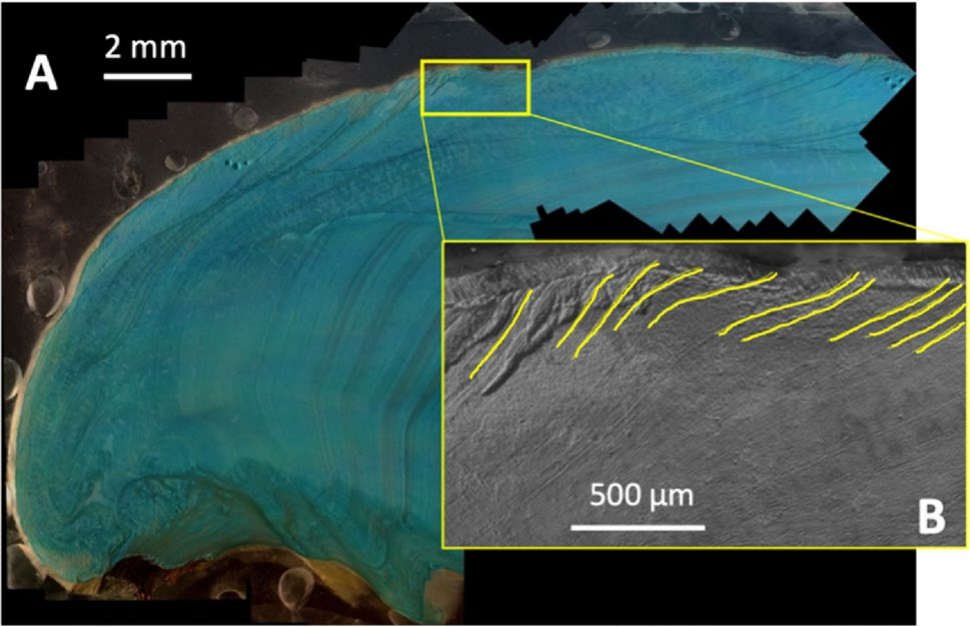
(**A**) Reflected light of H3R stained with Mutvei’s solution in 2.5X zoom. (**B**) Zoomed grayscale image of the yellow rectangle in image (**A**) with depictions of finer banding patterns along the ventral margin.

Growth rate chronologies were established using T(Δ_47_) and high-resolution growth band analyses from thin sections stained and etched with Mutvie’s solution (^51^; Fig. 5). Fine banding patterns were counted near the center portion of the shells where band width was more uniform, and time could be considered equivalent to band width. The monthly boundaries determined near the center of the shell were then traced throughout the shell to the ventral margin and growth rates were estimated by comparing the measured length of the shell from a stationary point on the umbo to the ventral margin at each monthly boundary marker. Additional description of band counting can be viewed in the method and supplemental sections. In the modern shell (3R5), growth is highest in the late fall and summer and ranges from 0.4 to 2.6 mm per month (Fig. 3A). Growth rates in the historic shell (H3R) are highest in late spring and summer and range from 0.3 to 2.2 mm per month (Fig. 4A). Reconstructed δ^18^O_water_ values are within measured mean monthly values (− 5.2‰ to 0.4‰; 33) and range from − 4.7 ± 0.8‰ to − 0.2 ± 0.7‰ and − 4.9 ± 1.1‰ to − 0.5 ± 0.7‰ for 3R5 (Fig. 3B) and H3R (Fig. 4B), respectively.

**Figure 6:**
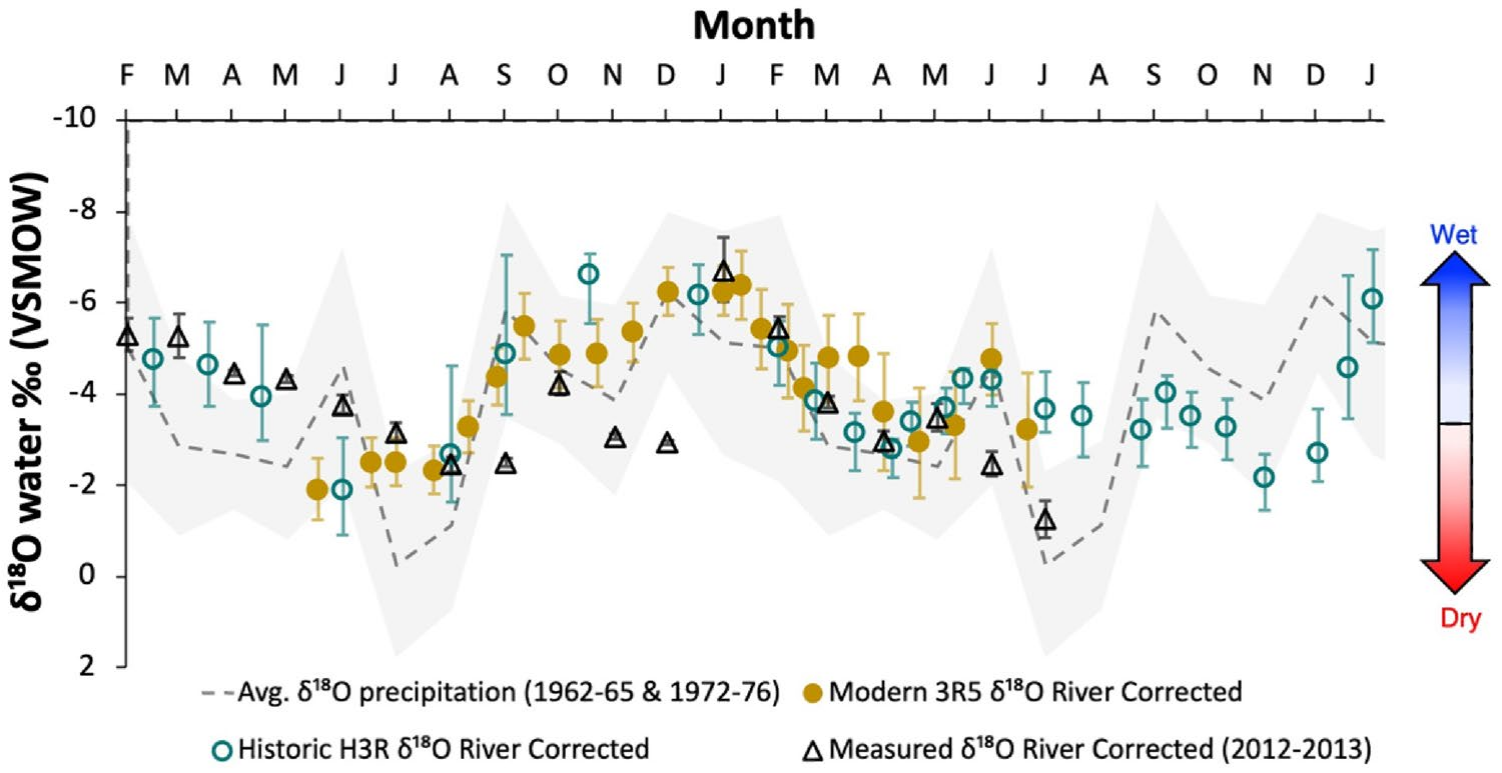
Comparison of Δ_47_-derived δ^18^O_water_ values after applying the correction of 1.7‰ to account for evaporation effects on the Brazos River water for modern shell 3R5 (filled yellow circles), historic shell H3R (teal open circles), measured river samples from Van Plantinga and Grossman (2017; black open triangles), and average δ^18^O of precipitation samples from Waco, TX during 1962–65 and 1972–76 (light grey dashed line, with minima and maxima bounded by the grey shaded area). All error bars represent the standard error. The blue and red shaded arrow on the right indicates direction of environmental conditions (wet = precipitation and dry = drought).

Comparisons of our reconstructed water values are shown in Fig. 6, where the baseline trend for precipitation, or meteoric water, was established using precipitation data collected from 1962 to 1965 and 1972 to 1976 in Waco, TX, ~ 148 km upstream of the mussel collecting site^52^. Reconstructed isotope values from H3R and 3R5, and the measured water values from 2012 to 2013 were corrected for the evaporation effect on the Brazos River water by subtracting 1.7‰ based on the difference between the mean ^18^O-enrichment in the Brazos River and the weighted mean δ^18^O of precipitation determined by Van Plantinga and Grossman^40^. This correction makes the comparison of our reconstructed values directly comparable to the measured values of precipitation values.

## Discussion

We interpret the isotopic record of mussel growth within the framework of mussel life cycles. Freshwater mussels have multi-stage life cycles, transitioning through the fertilized egg, glochidia, juvenile, and adult stages, respectively. *A. plicata* is a short-term brooder that spawns in late spring to early summer and releases glochidia a few weeks to a month later. Juvenile mussels allocate more energy towards growing and have higher growth rates before slowing as they reach sexual maturity at the age of 3 to 9 years^19^. Growth generally follows a logistic trend that eventually reaches an asymptotic length where growth becomes very slow or ceases. The faster growth rates of juvenile mussels can be rather plastic depending on life history and the surrounding environment^16,19^, but in general the faster growth rates make juvenile shell the best for detailed environmental records utilizing isotopic analyses. Specifically, clumped isotope measurements require large sample sizes (~ 1.2 mg) compared with oxygen isotopes (0.05 mg), so slower growth in the adult proportion of the shell would lead to a higher degree of averaging and less detailed environmental records.

In temperate latitudes, 23.5 to 66.5 degrees, freshwater mussel growth has typically been thought to slow and cease in the fall when temperatures reach below 6–12 °C and begin again the following spring when temperatures reach ~ 12–15 °C^4^; thus, peak growth occurs during spring and summer when temperatures are optimal (26–28 °C;^53^). However, in subtropical latitudes like our study area, seasonal temperature range is lower, which can extend the growing season and minimize growth cessation^43^. Haag et al.^54^ found that in streams across Kentucky, the onset of growth occurred at 17.8 °C. This prediction is higher than previously determined values of ~ 12–15 °C^55,56^ but may have been due to variation between study environments since growth is not solely temperature dependent. Instead, growth can be dependent on multiple factors including nutrient availability, alkalinity, temperature, latitude, and life stage^54,57,58^.

At our study site, average monthly air temperature is considered equivalent to water temperature plus or minus 1–2 °C^44^. Freshwater mussel growth is typically thought to slow or cease during winter when water temperatures reach below 6–12 °C and begin again the following spring when temperatures reach ~ 12–15 °C^4^. Mean air temperatures near the study site reach below 12 °C during the months of December and January but can experience average daily highs up to 16.5 °C and 17.4 °C, respectively. These average highs are not much lower than the predicted value for the onset of growth (~ 17.8 °C) determined by Haag et al.^54^ and higher than previously determined ranges (~ 12–15 °C) that can trigger the onset of growth. Therefore, we cannot confidently assume a complete cessation of growth from December to January, and instead assume slower growth at these lower temperatures. Based on our understanding of the study site and local temperature trends, we assume that the lowest calculated Δ_47_ temperatures occur in January and that Δ_47_ temperature follow the seasonal trend for air temperature based on monthly norms from NOAA climatological reports (i.e., means for 1991 to 2020 and the air–water temperature relationship for the Brazos River^44,45^).

For the modern shell (3R5), we determined the years sampled by the isotope analyses by first estimating the age of the specimen (~ 4 years) based on criteria described in Haag and Commens-Carson^17^, then subtracted the number of dark bands characteristic of annual bands within the sampled area from known date of collection. Since 3R5 was collected in August 2013, this yielded an age for isotopic samples of summer 2009 to summer 2010. Based on comparisons with local precipitation and discharge records, we find that the dark bands that fail to extend to the exterior of the shell align with periods of high river discharge and precipitation (Fig. 3C). During elevated stream flows, increased turbidity and sediment suspension can displace or stress mussels and lead to dissolution or precipitation misalignments that cause deposition of dark “disturbance bands” as the organism’s valves close and reopen^9,17,59,60^.

Reconstructed δ^18^O_water_ values in 3R5 for the period of high rainfall and increased natural discharge from mid-August to late-September (2009) decrease from − 0.6 to − 3.8‰ (Fig. 3B), revealing a significant freshening signal with the increased amount of ^18^O-depleted rainwater discharging to the river. Measured Δ_47_ temperature also show a gradual decrease, most likely in response to the cooler rainfall entering the river and decreased residence time that the water has available to warm during faster streamflow^61–63^. After that, both reconstructed δ^18^O_water_ and T(Δ_47_) increase before declining in fall and winter. The second dark band, or annual band, in 3R5 also correlates with the onset of high discharge based on our chronology. However, the observed increase in discharge in the 2009–2010 winter does not correlate with an increase in measured local precipitation, implying the major increase in flow was a response to release from the upstream impoundment at Lake Whitney and more northern precipitation events (Fig. S9).

The temperature corresponding with the annual band during this time recorded the coldest temperature, 17 °C, but was still higher than the expected 11 °C and 15 °C winter minimum based on the seasonal trend for mean air temperature (Fig. S6) and available water (Fig. S7) temperature, respectively. Winter releases from impoundments are known to increase water temperatures when released from the bottom waters of reservoirs and elevate δ^18^O_water_ due to enhanced evaporation in impounded water^40,46,64^, which would contribute to the warmer water temperatures observed and the potential to lengthen peak growing seasons. Water temperatures in the Lake Whitney reservoir are typically thermally stratified with cooler bottom water throughout the year until the fall turnover in October–November when waters become isothermal and lasts until the following spring. During this isothermal period, the water becomes well mixed with depth and water temperatures follow the surface and air temperature^65,66^. However, bottom waters after overturning can become thermally insulated from cooler surface temperatures in winter and contribute to warmer downstream temperature that deviates from cooler air temperatures during Lake Whitney releases^44^.

For the historic shell, H3R, we could not define an exact age of the sampling period because the archive lists an imprecise collection date (~ 1880–1900), but we were able to define it as growing within an environment that predates the damming of the Brazos River. Despite the lack of impoundment, reconstructed δ^18^O_water_ values calculated from H3R were within the monthly mean of modern measured values and those predicted by 3R5. Disturbance bands in H3R also correlated with decreases in δ^18^O_water_ before and after the summer season, suggesting that increased rainfall and associated runoff inhibited growth. The timing of these events also validates our methods used to reconstruct the chronology since peak precipitation in the Brazos River basin occurs in May and September^42^. During H3R’s late spring rainfall event in Year 2, Δ_47_ temperature measured ~ 25 °C, while Δ_47_ temperature during the rainfall event at the summer-fall transition decreased to ~ 18 °C (Fig. 4C,D). In both H3R and 3R5, growth rates slowed during the initial increases in stream flow but rebounded afterwards, most likely due to increased availability of nutrients via runoff and increased particulate settling with decreased turbulence^67–69^.

To compare our reconstructed δ^18^O_water_ results to measured δ^18^O values of meteoric water, we corrected the reconstructed and measured δ^18^O_water_ to account for evaporation effect on the surface water, and created a baseline using precipitation data collected from 1962–1965 to 1972–1976 in Waco, TX, ~ 148 km upstream of the mussel collecting site^40,52^. Overall, our reconstructed δ^18^O_water_ values show the expected trend for δ^18^O in the river when meteoric water is the main source (Fig. 6). Mean measured δ^18^O_water_ from Van Plantinga and Grossman^40^ also followed the meteoric trend, except for fall and winter measurements in 2012 when drought conditions were present. Though our baseline relies on a small sample set due to limited number of measured δ^18^O in precipitation recorded near our study site, deviations from the meteoric baseline can be an indication for drought and flood conditions.

Growth trends for both shells agree with previous findings of high growth in spring and summer, but also confirm that rapid growth can occur well into late fall^4,55^. There is no significant difference between modern and historic growth rates (two tailed *t*-test, *p* > 0.05; Fig. S7). However, only the historic shell (H3R) shows a significant correlation between growth rate and both reconstructed δ^18^O_water_ and T(Δ_47_) (R = 0.67, *p* < 0.001, and R = 0.43, *p* = 0.046, respectively; Fig. 8C,D). The stronger correlation between growth and reconstructed δ^18^O_water_ suggests that historic growth is more sensitive to changes in δ^18^O_water_, or precipitation, than to temperature (*p* ≥ 0.05 for both H3R and 3R5). Of course, these results are based on only two specimens; nevertheless, they contribute valuable insight for understanding the factors driving community level changes in mussel populations within the Brazos River and reveal the potential of clumped isotopes in freshwater mussels as a proxy in rivers that experience flow alterations.

## Conclusions and Implications

This study successfully used clumped isotope analyses to reconstruct seasonal δ^18^O_water_ values for *A. plicata* from the Brazos River, located in Central Texas. Disturbance bands correlate with rainfall and high streamflow events based on reconstructed δ^18^O_water_ and known relationships between river water δ^18^O and precipitation. Calculated growth rates ranged from 0.3 to 2.5 mm per month with high growth in the spring through fall, suggesting an extended growing season. No significant relationship with growth rate was found in the modern shell (3R5), but the significant relationship between reconstructed δ^18^O_water_ and growth rates in the historic shell (H3R) suggests that precipitation and flow rate are stronger drivers than temperature in historic growth that predates river impoundment and represents natural flow conditions. The results from these two shells demonstrate that clumped isotope analyses can provide valuable understanding of local hydrologic cycles and climate events such as droughts and floods on multiyear time scales. However, broader application using museum, fossil, and live-collected specimens would make it possible to examine historic and prehistoric climate change over a wide swath of the US mid-continent, while at the same time revealing the impact of river impoundment and climate change on mussel growth and survival.

In addition to understanding the relationship between mussel growth, flow rates, and climate change, clumped isotope analyses can be helpful for validating growth and longevity estimates. This is important because these biological end points are often used to make inferences on how a species will respond to environmental disturbance or different management actions. For example, Randklev et al.^9^ evaluated how mussels cope with flow rates and found that species with high growth rates and reduced longevity were more tolerant of streambed disturbance during high flow events. In contrast, species with lower growth rates and longer lifespans were less resilient. Such inferences can be used to guide environmental flow recommendations^70^. However, it is important that age and growth estimates are validated to minimize inaccurate determinations that could lead to inappropriate management recommendations that are not protective during floods or droughts. This could be done by additional coupled isotope-growth band studies of shells from the Brazos and other rivers as an alternative to the time-consuming caged experiments that can create artificial growth hiatuses with each handling. The literature is replete with examples of the impact of inaccurate estimates for these endpoints. For example, the orange roughy (*Hoplostethus atlanticus*) was intensively harvested off New Zealand based on the presumption that it lived 20 to 30 years and had a fast growth rate. More recent research has shown that it is much longer lived, over 100 years, with a slow growth rate^71^.

For unionid mussels, examples of inaccurate determinations of age and growth and their impact on management actions are not as well documented. This is due, in part, to the fact that growth and longevity data are lacking for most mussel species. To date, these data are available for only 35 of the 300 mussel species known to occur in North America. For these species, very few if any have been validated and to the best of our knowledge clumped isotopes have only been used on two species (*Amblema plicata* and *Crytonias tampicoensis*^27^). Thus, expanding this knowledge base and validating age and growth estimates is critical for the long-term conservation of mussels as a group. Future efforts should focus not only on evaluating the ecology of different species but also on creating records spanning historic drought periods such as the Texas drought of record (1950–57) and deeper time (e.g., Pliocene warm period) with high temperatures and drought events that resemble predictions for future climate.

## Materials and methods

### Samples and sampling

Shell specimens were collected from the main stem of the middle Brazos River west of College Station, TX (30.55 latitude, − 96.42 longitude; Fig. 1) in 2013 and ~ 1900 (30.6 latitude, − 96.4 longitude; Fig. 1). The modern sample was collected live from the shoal water by the third author and the historic specimen was provided by the Singley Collection from the University of Texas at Austin Non-Vertebrate Paleontology Laboratory (collector unknown). Discharge data were compiled from the USGS gauge 08,108,700 near Bryan, TX^72^ and precipitation δ^18^O values for Waco, TX were retrieved from the Global Network of Isotopes in Precipitation (GNIP;^52^).

Upon arrival, shells were cleaned with double deionized water and the right valves were sectioned along the long axis of growth and then sequentially cut into thick (~ 2–3 mm) and thin sections (300–400 μm). Thin sections were then examined under the microscope for physical discoloration and damage that could indicate chemical alterations. A New Wave micromill equipped with a ~ 400 μm dental bur, operated at low speed to prevent heating and reordering of the clumped isotopes, was used to mill carbonate powders parallel to growth bands of juvenile growth (first 2–3 years) in 300–350 μm increments along the ventral margin of thick sections (Fig. 2).

### Isotope measurements

Isotope analyses were performed using a Kiel IV carbonate device coupled to a Thermo Fisher Scientific 253 + isotope ratio mass spectrometer (IRMS) in the Stable Isotope Geosciences Facility (SIGF) at Texas A&M University using methodology described in Meinicke et al.^73^ and Barney and Grossman^35^. We analyzed 5–10 replicates of each individual powdered sample weighing ~ 120 µm and used a running mean of three samples to obtain 15–30 replicates for each data point and increase precision. Analytical precisions (1σ) for ETH standards during the period of sample analyses were ± 0.03‰, ± 0.05‰, and 0.040‰ for δ^13^C, δ^18^O, and Δ_47_, respectively. For sample analyses of unknowns, δ^18^O_shell_, δ^13^C_shell_, and Δ_47_ uncertainties were calculated based on the standard error for each sample and its 5–10 replicates and then averaged for each three-sample mean (15–30 replicates) that define each data point. Errors for T(Δ_47_) and δ^18^O_water_ were estimated using the propagation of the minima and maxima of the Δ_47_ uncertainty (Table S1). Overall, the average standard error (SE) for measured Δ_47_ equates to ~  ± 3.5 °C. Clumped (Δ_47_) temperatures were calculated using the equation of Anderson et al.^74^:

1 $$\Delta {47} = 0.0391\left( { \pm \,0.0004} \right) \cdot \frac{{10^{6} }}{{T^{2} }} + 0.154\left( { \pm \,0.004} \right)$$

where *T* is the water temperature in kelvin and Δ_47_ is standardized within the “InterCarb-Carbon Dioxide Equilibrium Scale” (I-CDES). Reconstructed δ^18^O_water_ values were calculated using the rearranged δ^18^O paleotemperature equation from Grossman and Ku^26^ corrected for water δ^18^O in terms of VSMOW^75^:

2 $${\text{T}}\left( {^\circ {\text{C}}} \right) = 19.7 - 4.34\left( {\updelta^{18} {\text{O}}_{{{\text{aragonite }}\left( {{\text{VPDB}}} \right)}} - \updelta^{18} {\text{O}}_{{{\text{water }}\left( {{\text{VSMOW}}} \right)}} } \right)$$

### Chronologies and growth rates

Monthly chronologies were assigned using a combination of the Δ_47_ temperature, shell banding, and monthly air temperature norms, or monthly averages over a 30-year period, for College Station based on NOAA records. Preliminary chronologies were determined using the shell length at individual milling transects and the local trend for monthly air temperature norms. The shell intervals with the coolest Δ_47_ temperatures were assigned to the month of January, while the interval with the maximum Δ_47_ temperature was assigned to the month of July, matching the local minimum and maximum of average seasonal air temperatures. This preliminary chronology was then refined using thin sections stained with Mutvei’s solution to enhance the fine banding patterns and better resolve the distance between annual bands and monthly boundaries (Figs. S1 and S2;^51^). Sub-annual bands were counted in the center of the shell near the beak area where bands had almost uniform band width. Assuming equal bandwidth and time, we subdivided the sub-annual growth bands between the previously determined temperature minima and maxima within a years’ time, or area between two annual bands. The monthly boundaries between sub-annual bands in the center of the shell were then traced throughout the shell and marked on scan images of each shell thin section. Monthly growth rates were then calculated by measuring the difference in shell length from the umbo to the edge of the ventral margin at the assigned monthly boundaries. See supplemental material for further discussion on chronology anchoring and sub-annual banding for the individual shells.

### Supplementary Information

Supplementary Information.

## Data Availability

Data are provided in the supplemental material and more detailed methods and raw data will be made available upon request from the authors M.A. Brewer and E.L. Grossman.
