# Supplementary material for: Clumped isotopes reveal relationship between mussel growth and river discharge
Source: Sci Rep. 2024 Apr 1;14:7653. doi: 10.1038/s41598-024-58246-w (PMC11369190; doi:10.1038/s41598-024-58246-w)
Supplement: Supplementary file 1 — Supplementary Information. [file 41598_2024_58246_MOESM1_ESM.pdf]

Clumped isotopes reveal relationship between mussel growth and river discharge

Melanie A. Brewer[1], Ethan L. Grossman[1], & Charles R. Randklev[2]

[1]*Department of Geology and Geophysics, Texas A&M University, College Station, TX, 77843, USA*
[2]*Texas A&M Natural Resources Institute, Dallas, TX, 75252, USA*

E-mail: e-grossman@tamu.edu

SUPPLEMENTAL MATERIALS

Chronology Anchoring and Sub-annual Banding

Shell chronologies were defined using a two-step procedure in which rough chronologies were first based on measured clumped temperatures (T(Δ_{47})) and distance along the shell for each isotope sample, followed by revised chronologies based on sub-annual banding. Preliminary chronologies were anchored using the coolest and warmest Δ_{47} values, which were assigned to January and July, respectively (see Figure S4). These cold and warm temperatures are marked by the blue and red lines, respectively, on Figures S1 and S2. Following the preliminary chronologies, thin sections were stained and etched using Mutvie's solution for 25 minutes in a continuously stirred solution held at ~37-40℃. Immediately after removing the microscope slide from the solution, it was gently rinsed with double deionized water and set aside to air dry. To count sub-annual growth bands, the center of shells near the beak and umbo were targeted because bands had nearly uniform thickness that would help to define time equally before bands began to widen as they reached the outer portions of the shell. In the figures below, this region is marked by the yellow outlined regions. At these areas, individual bands were visible under 2.5X magnification using standard microscopy with an external light reflected obliquely across the thin section surface. Bands were counted and marked with green lines on Figures S1 and S2. For the modern shell (3R5) a total of 97 bands were counted, while the historic shell (H3R) had 168 bands within the sampled area. Since band widths were similar across most of the targeted transects, we divided the area between the anchored cold and warm markers into equal segments to represent the boundaries between months. These monthly boundaries are representing as white markers on the figures. Note that some of the monthly markers overlap the sub-annual band markers and some fall in the middle of a sub-annual band. Monthly boundaries, or solid white lines, that do not overlap a sub-annual band boundary were not counted towards the overall band count. The estimated monthly boundaries in the center of the shells were then traced along the shell and marked on the ventral margin of the shells with the corresponding line color. Monthly growth rate chronologies where then calculated using these monthly boundaries by measuring the length from a stationary point on the edge of the umbo to the boundary of the ventral margin. Software used to stitch together thin section images and measure length across the shell include Microsoft Image Composite Editor (ICE 2.0) 64-bit and ImageJ v.1.53a, respectively.

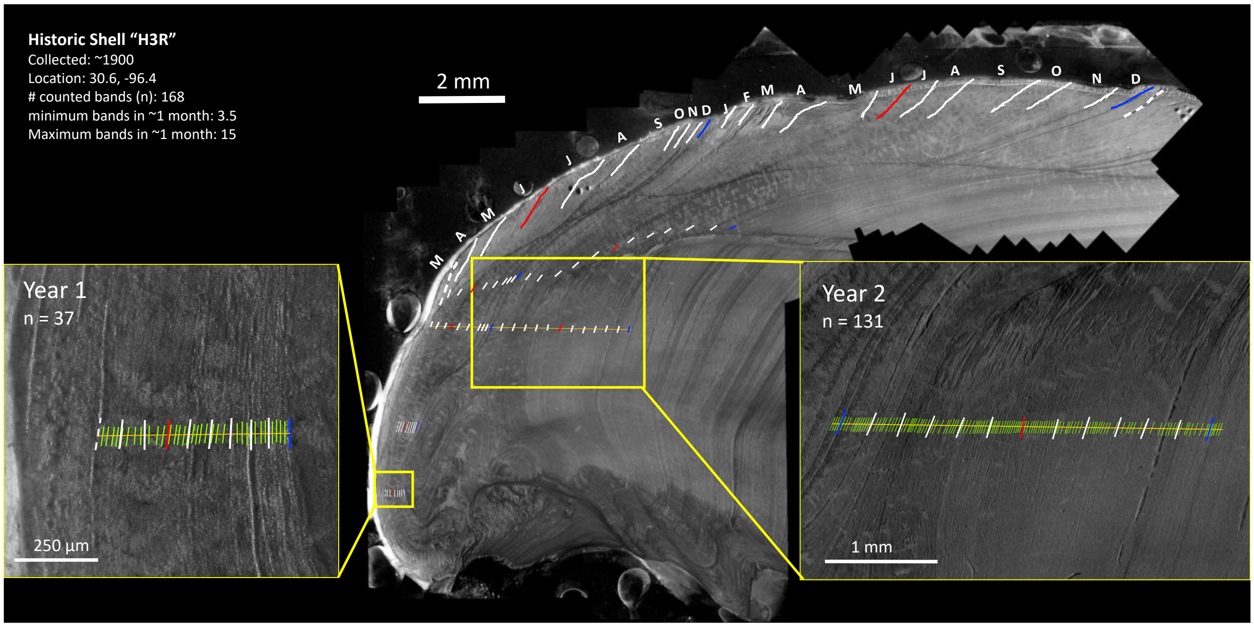

Figure S1. Greyscale image of the stained and etched historic shell "H3R". Areas targeted for best visibility and nearly uniform band width are outlined in the yellow boxed regions. Boundaries between sub-annual bands are marked by the green lines in the yellow boxes. Blue and red line markers represent the T(Δ_{47}) anchored samples, and the white solid lines represents additional month boundaries. White dashed lines represent the beginning and ending of the entire sampling area. Across the entire sampling area, 168 sub-annual bands were counted, which equates to ~2-day resolution on average and ~8-day to 2-day resolution between individual monthly markers. The mean, minimum and maximum sub-annual band widths were 21.8 µm, 6.1 µm, 64.9 µm, respectively. The mode and median were 16.2 µm and 19.3 µm, respectively.

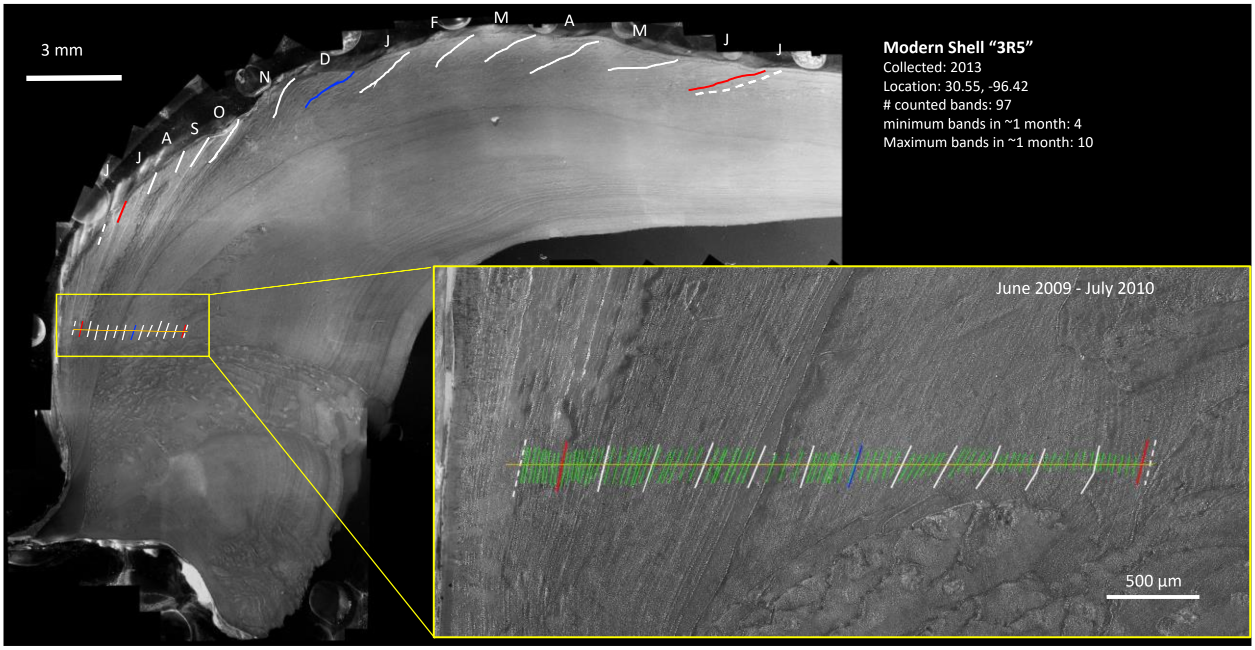

Figure S2. Greyscale image of the stained and etched modern shell "3R5". Areas targeted for best visibility and nearly uniform band width are outlined in the yellow boxed regions. Boundaries between sub-annual bands are marked by the green lines in the yellow boxes. Blue and red line markers represent

the T(Δ_{47}) anchored samples, and the white solid lines represents additional month boundaries. White dashed lines represent the beginning and ending of the entire sampling area. Across the entire sampling area, 97 sub-annual bands counted, which equates to ~4-day resolution on average and weekly to 3-day resolution between individual monthly markers. The mean, minimum and maximum sub-annual band widths were 30.2 μm, 5.6 μm, and 77.3 μm, respectively. The mode and median were both 30.5 μm.

Table S1. Isotope measurements, Δ_{47} temperatures (Anderson et al., 2021), and calculated $\delta^{18}O_{water}$ (Grossman and Ku, 1986; Hudson and Anderson, 1989) with reconstructed dates.

Sample	Reconstructed Date	$\delta^{13}C$ (VPDB)	$\delta^{13}C$ SE	$\delta^{18}O$ (VPDB)	$\delta^{18}O$ SE	T ℃ (I-CDES)	+ T ℃ SE	- T ℃ SE	$\delta^{18}O_{water}$ (VSMOW)	+ $\delta^{18}O_{water}$ SE (VSMOW)	- $\delta^{18}O_{water}$ SE (VSMOW)
3R5-1	1-Jun	-6.89	0.90	-2.22	0.33	27.5	2.9	2.9	-0.22	0.68	0.66
3R5-2	1-Jul	-7.78	0.64	-1.77	0.13	23.0	2.3	2.4	-0.81	0.55	0.54
3R5-3	15-Jul	-8.91	0.51	-1.34	0.40	21.1	2.3	2.3	-0.82	0.53	0.52
3R5-4	5-Aug	-10.07	1.12	-1.38	0.43	22.1	2.3	2.3	-0.63	0.53	0.52
3R5-5	25-Aug	-10.90	0.64	-2.09	0.89	21.0	2.4	2.5	-1.58	0.58	0.56
3R5-6	10-Sep	-10.56	0.93	-3.19	0.58	21.1	2.6	2.7	-2.68	0.62	0.60
3R5-7	25-Sep	-8.96	0.98	-3.93	0.17	19.5	3.0	3.1	-3.79	0.72	0.70
3R5-8	15-Oct	-7.48	0.76	-4.10	0.10	22.9	3.2	3.3	-3.17	0.76	0.73
3R5-9	5-Nov	-6.21	0.65	-4.30	0.11	23.7	3.1	3.2	-3.19	0.75	0.72
3R5-10	25-Nov	-5.89	0.41	-4.40	0.12	22.1	2.8	2.9	-3.66	0.66	0.64
3R5-11	15-Dec	-6.43	0.81	-4.40	0.12	18.2	2.3	2.3	-4.56	0.53	0.52
3R5-12	15-Jan	-7.01	0.45	-4.27	0.13	17.6	2.3	2.4	-4.55	0.55	0.54
3R5-13	25-Jan	-6.96	0.49	-4.27	0.13	17.0	3.2	3.4	-4.69	0.77	0.75
3R5-14	5-Feb	-6.31	0.26	-4.47	0.18	20.0	3.7	3.9	-4.19	0.89	0.86
3R5-15	20-Feb	-5.96	0.13	-4.55	0.11	24.4	4.4	4.6	-3.26	1.05	1.01
3R5-16	1-Mar	-5.62	0.24	-4.48	0.14	27.7	3.9	4.1	-2.44	0.94	0.90
3R5-17	15-Mar	-5.27	0.25	-4.26	0.09	23.9	4.0	4.2	-3.10	0.96	0.92
3R5-18	1-Apr	-5.19	0.18	-3.96	0.25	22.5	4.0	4.2	-3.12	0.97	0.93
3R5-19	15-Apr	-5.74	0.56	-3.71	0.19	26.6	5.4	5.7	-1.93	1.31	1.24
3R5-20	5-May	-6.62	0.59	-3.73	0.21	29.5	5.1	5.4	-1.26	1.24	1.17
3R5-21	25-May	-7.57	0.46	-3.98	0.22	29.0	5.0	5.2	-1.64	1.20	1.14
3R5-22	15-Jun	-8.16	0.31	-4.24	0.05	23.9	3.3	3.4	-3.07	0.79	0.76
3R5-23	5-Jul	-8.02	0.45	-4.19	0.10	30.3	5.2	5.5	-1.54	1.27	1.21
**Mean**		**-7.33**	**0.54**	**-3.62**	**0.22**	**23.3**	**3.4**	**3.6**	**-2.64**	**0.82**	**0.79**
**Minimum**		**-10.90**		**-4.55**		**17.0**			**-4.69**		
**Maximum**		**-5.19**		**-1.34**		**30.3**			**-0.22**		
H3R-1	1-Mar	-6.72	0.24	-2.50	0.10	16.4	4.2	4.4	-3.05	1.02	0.97
H3R-2	1-Apr	-6.89	0.20	-2.77	0.16	18.1	3.8	4.0	-2.94	0.91	0.88
H3R-3	1-May	-7.01	0.13	-2.97	0.10	22.0	4.0	4.1	-2.23	0.95	0.92
H3R-4	15-Jun	-6.68	0.16	-2.96	0.10	30.8	4.1	4.3	-0.20	0.99	0.95
H3R-5	15-Aug	-6.72	0.17	-3.88	0.35	31.4	4.4	4.6	-0.98	1.06	1.01
H3R-6	15-Sep	-7.27	0.29	-4.78	0.44	25.8	5.4	5.7	-3.17	1.32	1.24
H3R-7	1-Nov	-8.24	0.12	-5.46	0.20	21.1	4.6	4.8	-4.93	1.10	1.05
H3R-8	1-Jan	-8.65	0.13	-4.70	0.17	19.8	3.7	3.9	-4.48	0.90	0.86
H3R-9	15-Feb	-8.85	0.06	-4.09	0.12	22.2	3.5	3.7	-3.32	0.84	0.81
H3R-10	8-Mar	-8.73	0.08	-3.35	0.15	24.0	3.6	3.7	-2.16	0.85	0.82
H3R-11	30-Mar	-8.21	0.14	-2.86	0.13	24.9	3.5	3.6	-1.46	0.83	0.80
H3R-12	20-Apr	-7.51	0.16	-2.61	0.03	25.4	2.6	2.7	-1.10	0.62	0.61
H3R-13	1-May	-6.91	0.08	-2.92	0.07	24.1	2.3	2.3	-1.71	0.53	0.52
H3R-14	20-May	-6.82	0.06	-3.43	0.09	25.1	2.3	2.4	-1.99	0.55	0.54
H3R-15	30-May	-7.19	0.11	-3.91	0.08	24.4	2.3	2.4	-2.63	0.54	0.53
H3R-16	15-Jun	-7.78	0.13	-3.93	0.09	24.6	2.4	2.4	-2.61	0.56	0.55
H3R-17	15-Jul	-8.05	0.09	-3.45	0.17	25.3	2.2	2.2	-1.96	0.51	0.50
H3R-18	20-Aug	-8.42	0.11	-3.02	0.11	23.9	3.7	3.8	-1.86	0.88	0.85
H3R-19	7-Sep	-8.13	0.14	-2.82	0.04	24.3	3.4	3.6	-1.57	0.82	0.79
H3R-20	21-Sep	-7.90	0.11	-2.83	0.02	20.8	3.3	3.4	-2.38	0.78	0.76
H3R-21	5-Oct	-7.49	0.03	-2.52	0.08	21.9	2.9	3.0	-1.82	0.69	0.67
H3R-22	25-Oct	-7.56	0.05	-1.99	0.13	20.6	3.1	3.2	-1.58	0.73	0.71
H3R-23	15-Nov	-7.71	0.05	-1.59	0.08	23.8	3.0	3.1	-0.46	0.71	0.69
H3R-24	15-Dec	-7.81	0.04	-2.16	0.28	23.8	2.7	2.8	-1.02	0.65	0.63
H3R-25	1-Jan	-8.17	0.08	-3.20	0.25	20.2	4.6	4.8	-2.88	1.11	1.06
H3R-26	15-Jan	-8.41	0.09	-3.91	0.06	17.0	4.0	4.2	-4.33	0.96	0.92
**Mean**		**-7.69**	**0.12**	**-3.25**	**0.14**	**23.1**	**3.4**	**3.6**	**-2.26**	**0.82**	**0.82**
**Minimum**		**-8.85**		**-5.46**		**16.4**			**-4.93**		
**Maximum**		**-6.68**		**-1.59**		**31.4**			**-0.20**		

Note: VPDB – Vienna Peedee belemnite, I-CDES – InterCarb-Carbon Dioxide Equilibrium Scale at 90°C, SE – standard error, and VSMOW – Vienna Standard Mean Ocean Water.

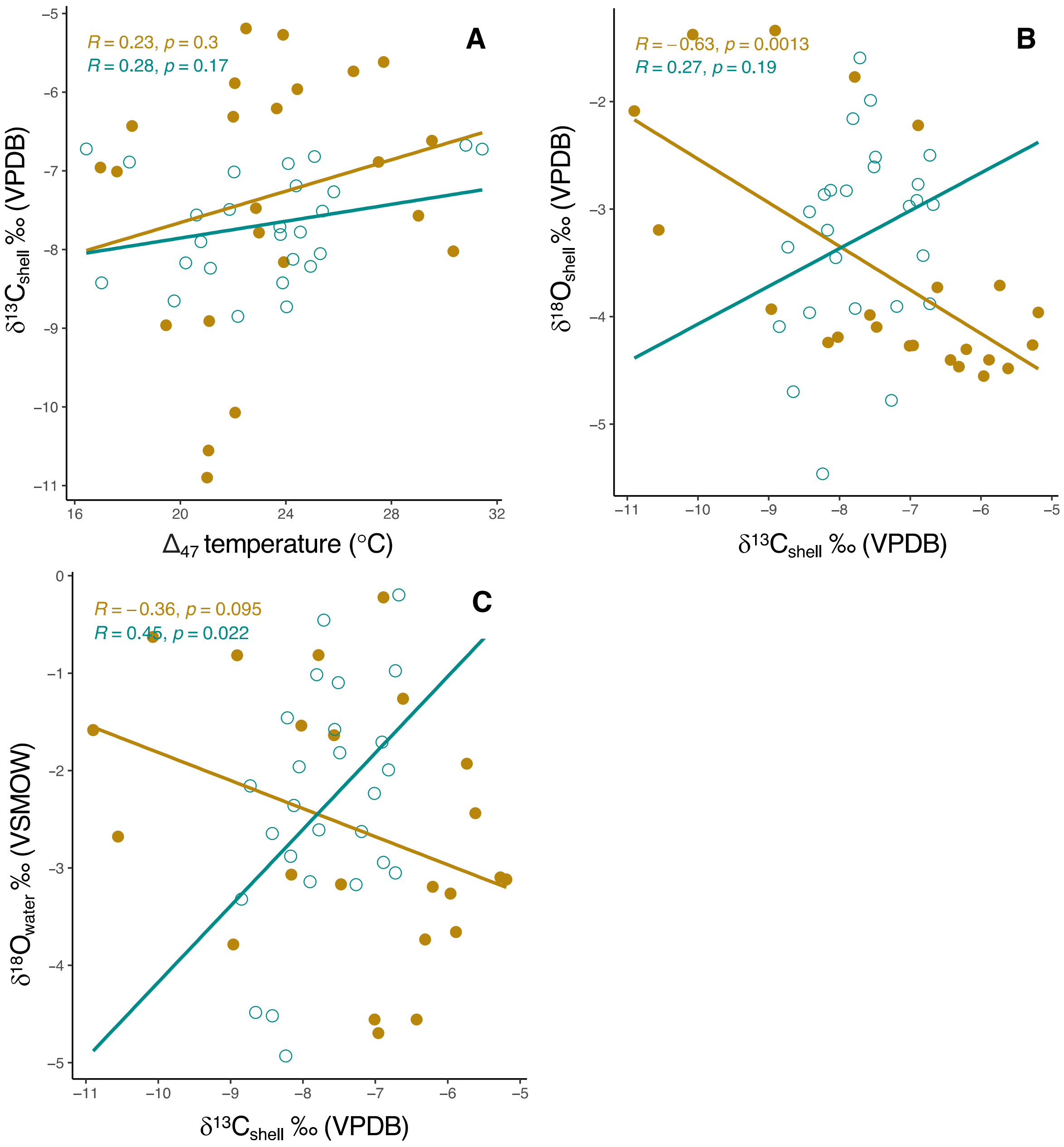

Figure S3. X-Y plot of (**A**) Δ_{47} temperature vs $\delta^{13}C_{shell}$, (**B**) $\delta^{13}C_{shell}$ versus $\delta^{18}O_{shell}$, and (**C**) $\delta^{13}C_{shell}$ versus reconstructed $\delta^{18}O_{water}$. The historic shell (H3R) and modern shell (3R5) plotted as open teal circles and filled gold circles, respectively. Pearson R correlations and *p*-values are recorded in the top left corner.

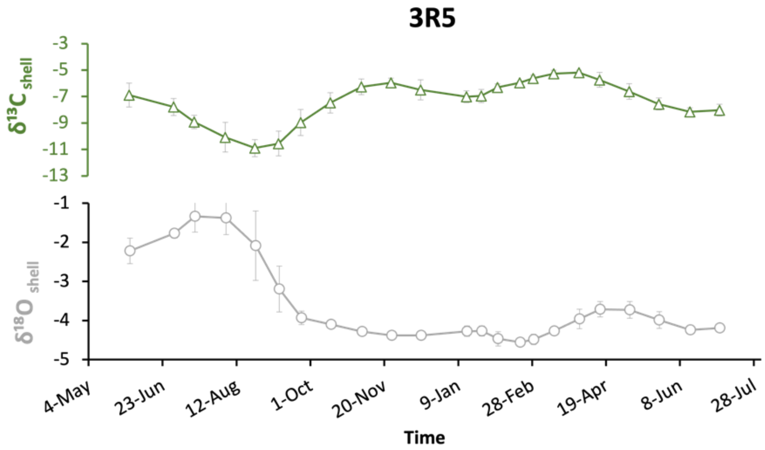

Figure S4. $\delta^{18}O$ and $\delta^{13}C$ records for modern shell (3R5) plotted relative to reconstructed chronology.

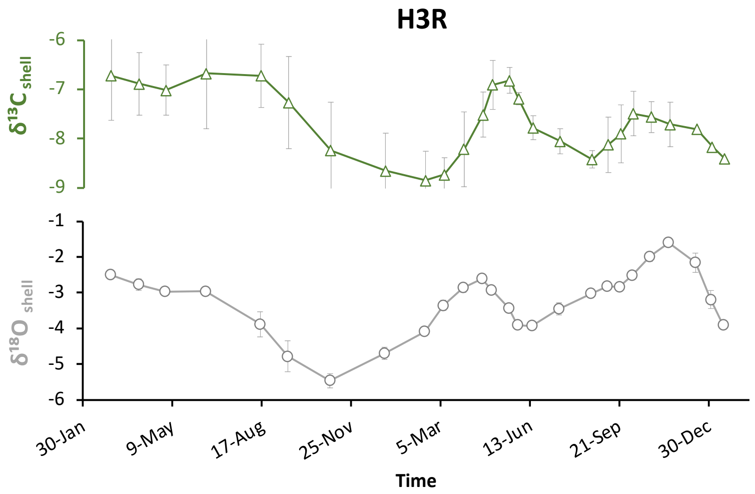

Figure S5. $\delta^{18}O$ and $\delta^{13}C$ records for historic shell (H3R) plotted relative to reconstructed chronology.

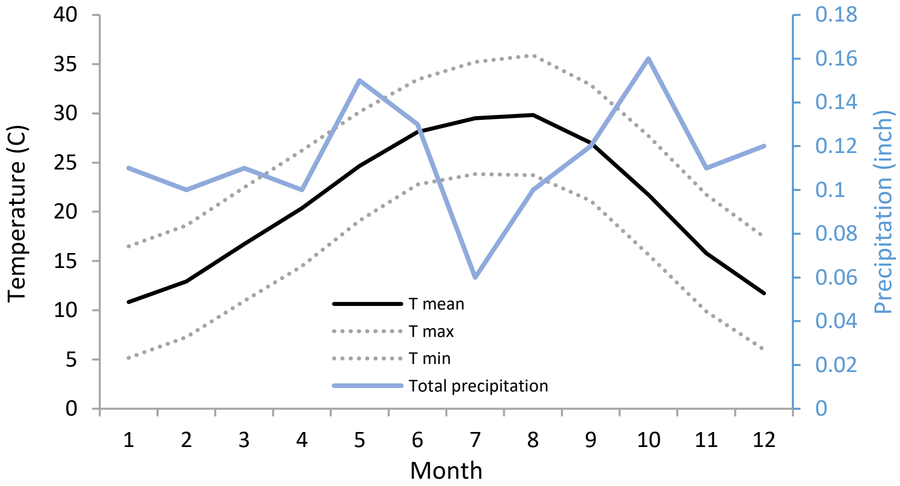

Figure S6. Plot of the monthly means for temperature (black) and precipitation (blue) during the period of 1961 to 2020. Dotted grey lines represent the maximum and minimum values for temperature.

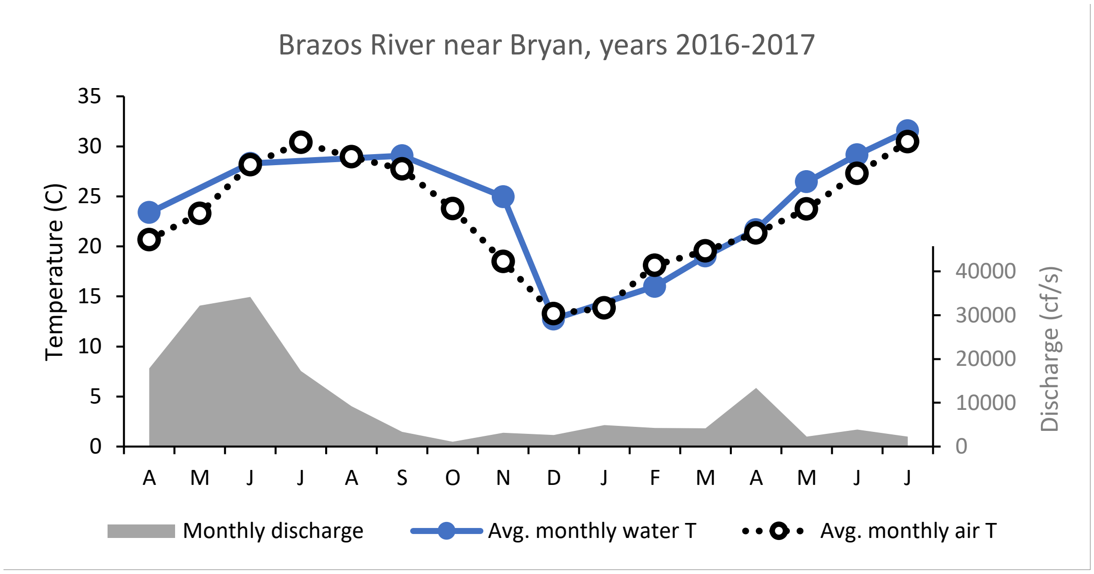

Figure S7. Plot of the average monthly air temperature (dotted black) for College Station, TX., water temperature (solid blue), and discharge (grey shading) from the Brazos River gauge near Bryan, TX. Data w collected from usgs.gov for all years that water temperature was recorded, which was from 2016 to 2017.

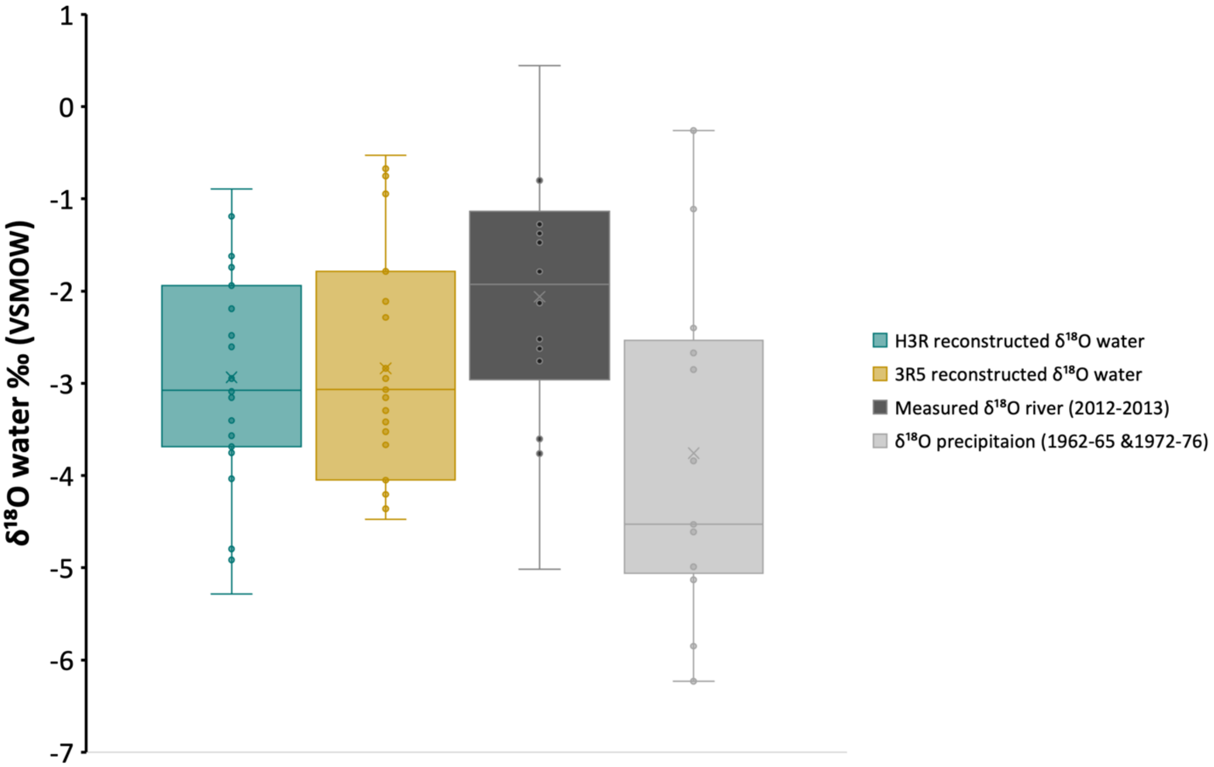

Figure S8. Box and Whisker plot for the δ^{18}O of reconstructed H3R (teal) and 3R5 (yellow) waters, measured water (dark grey), and precipitation (light grey). The “x” in the boxes represents the mean and the line within the box represent the median. The whiskers represent the max and min values. The top edge of the box represents the 3rd quartile, and the lower edge represents the 1st quartile.

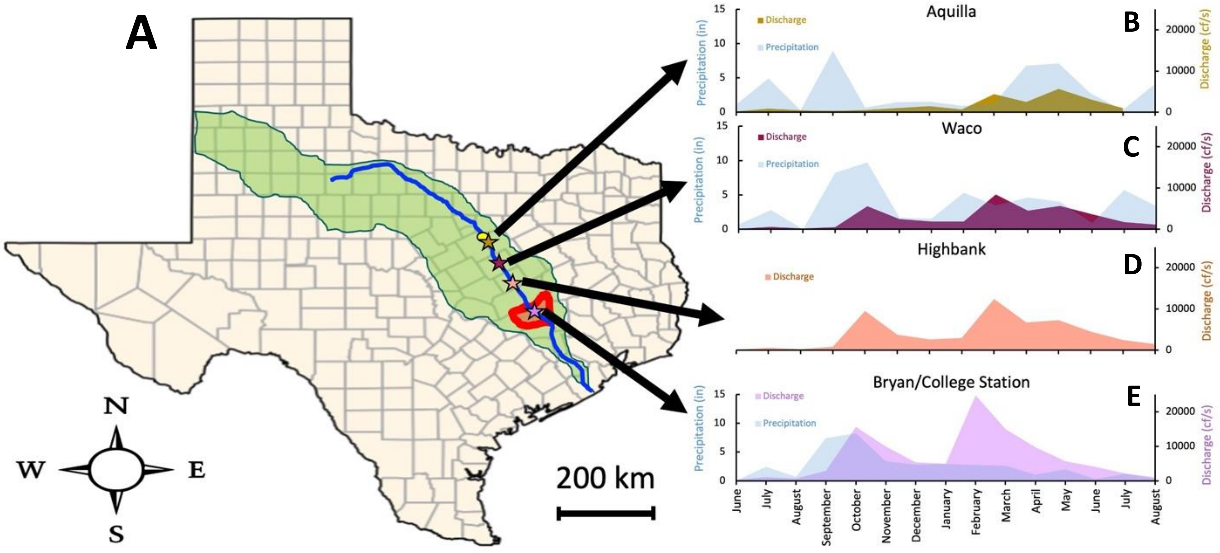

Figure S9. Mean monthly discharge and total precipitation profiles from June 2009 to July 2010 for the stretch of the Brazos River directly below the Lake Whitney Dam (yellow circle) and the sampling area (light purple star). **(A)** Map of Texas with the Brazos River (blue line) and Brazos drainage basin (green shaded area) showing the gauge location for the Aquilla (gold star), Waco (dark magenta star), Highbank (salmon star), and Bryan/College Station (light purple star) sites. **(B-E)** Profiles of the precipitation (light blue) and discharge (color follows that in A) along the river from the most upstream gauge on top to the more downstream gauges moving downward. *Note: Precipitation records were not available for the Highbank location during this time. The Texas map was created by the first author using the* tidyverse *and* map_data[in ggplot2] *packages* in R v4.3.1 *and* Rstudio v2023.-9.1+494. *Secondary layers and points for the drainage basin, river path, dam locations, and study area were added in Microsoft PowerPoint v16.81.*

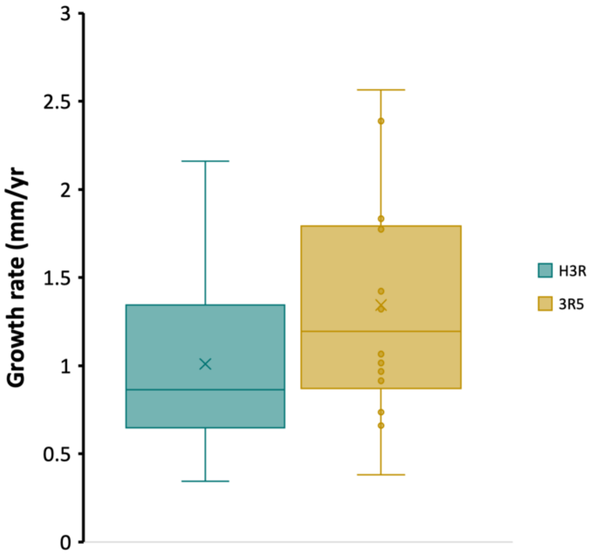

Figure S10. Box and whisker plot for growth rates in historic shell H3R (teal) and modern shell 3R5 (yellow). The “x” in the boxes represents the mean and the line within the box represents the median. The whiskers represent the max and min values. The top edge of the box represents the 3rd quartile, and the lower edge represents the 1st quartile.

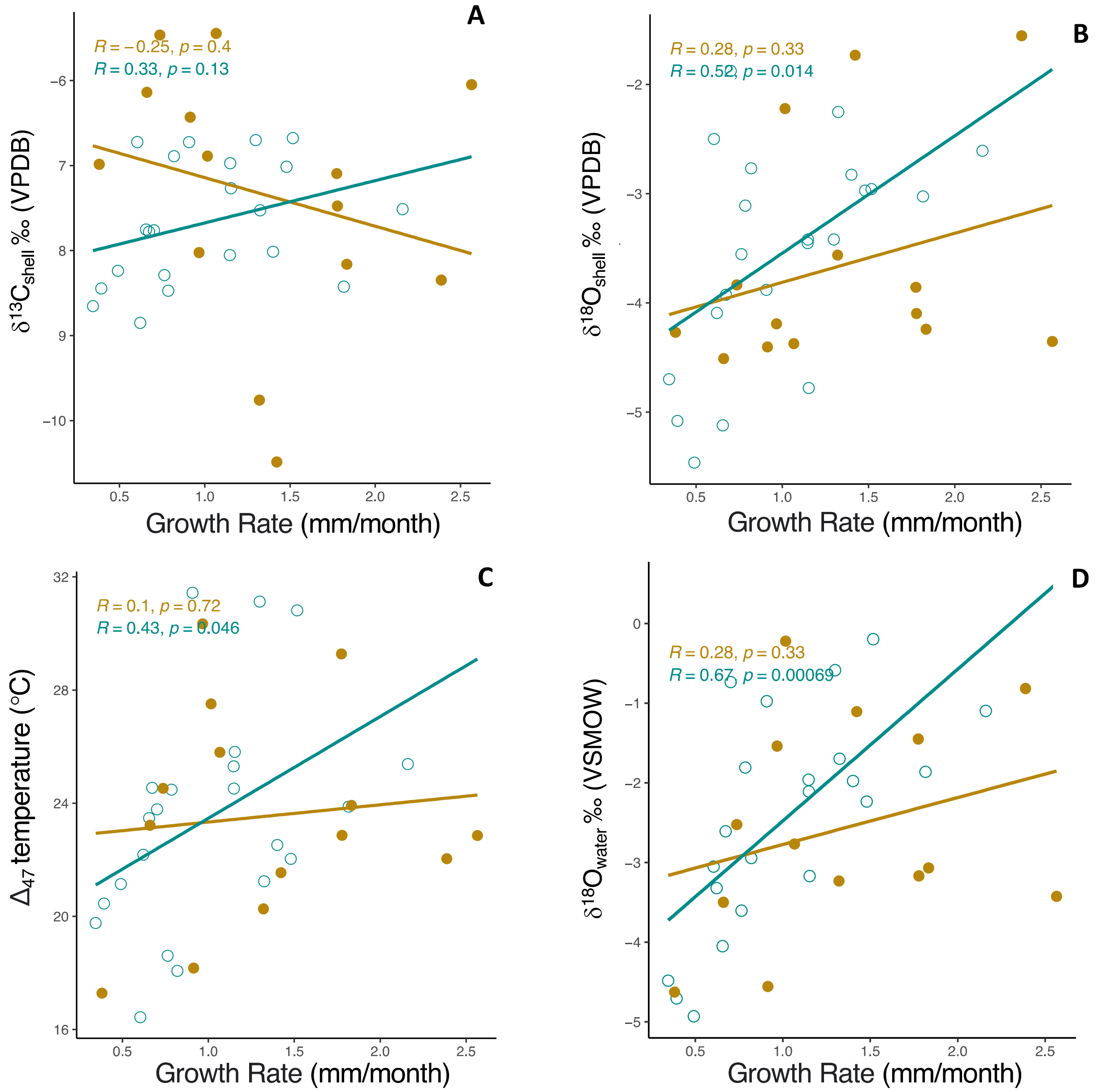

Figure S11. X-Y plot of monthly growth rate versus the monthly averages of (**A**) $\delta^{13}C_{shell}$, (**B**) $\delta^{18}O_{shell}$, (**C**) Δ_{47} temperature, and (**D**) $\delta^{18}O_{water}$. Historic shell (H3R) and modern shell (3R5) are plotted as open teal circles and filled gold circles, respectively. Pearson R correlations and *p*-values are recorded in the top left corner.
